# Performance Assessment of Two Low-Cost PM_2.5_ and PM_10_ Monitoring Networks in the Padana Plain (Italy)

**DOI:** 10.3390/s24123946

**Published:** 2024-06-18

**Authors:** Giovanni Gualtieri, Lorenzo Brilli, Federico Carotenuto, Alice Cavaliere, Tommaso Giordano, Simone Putzolu, Carolina Vagnoli, Alessandro Zaldei, Beniamino Gioli

**Affiliations:** 1 National Research Council, Institute of Bioecomony (CNR-IBE), Via Caproni 8, 50145 Firenze, Italy; lorenzo.brilli@ibe.cnr.it (L.B.); federico.carotenuto@ibe.cnr.it (F.C.); tommaso.giordano@ibe.cnr.it (T.G.); simone.putzolu@ibe.cnr.it (S.P.); carolina.vagnoli@ibe.cnr.it (C.V.); alessandro.zaldei@ibe.cnr.it (A.Z.); beniamino.gioli@cnr.it (B.G.); 2 National Research Council, Institute of Polar Sciences (CNR-ISP), Via P. Gobetti 101, 40129 Bologna, Italy; alice.cavaliere@cnr.it

**Keywords:** air quality, low-cost sensor, PM_2.5_, PM_10_, PurpleAir, AirQino, Padana Plain

## Abstract

Two low-cost (LC) monitoring networks, PurpleAir (instrumented by Plantower PMS5003 sensors) and AirQino (Novasense SDS011), were assessed in monitoring PM_2.5_ and PM_10_ daily concentrations in the Padana Plain (Northern Italy). A total of 19 LC stations for PM_2.5_ and 20 for PM_10_ concentrations were compared vs. regulatory-grade stations during a full “heating season” (15 October 2022–15 April 2023). Both LC sensor networks showed higher accuracy in fitting the magnitude of PM_10_ than PM_2.5_ reference observations, while lower accuracy was shown in terms of RMSE, MAE and R^2^. AirQino stations under-estimated both PM_2.5_ and PM_10_ reference concentrations (MB = −4.8 and −2.9 μg/m^3^, respectively), while PurpleAir stations over-estimated PM_2.5_ concentrations (MB = +5.4 μg/m^3^) and slightly under-estimated PM_10_ concentrations (MB = −0.4 μg/m^3^). PurpleAir stations were finer than AirQino at capturing the time variation of both PM_2.5_ and PM_10_ daily concentrations (R^2^ = 0.68–0.75 vs. 0.59–0.61). LC sensors from both monitoring networks failed to capture the magnitude and dynamics of the PM_2.5_/PM_10_ ratio, confirming their well-known issues in correctly discriminating the size of individual particles. These findings suggest the need for further efforts in the implementation of mass conversion algorithms within LC units to improve the tuning of PM_2.5_ vs. PM_10_ outputs.

## 1. Introduction

According to the latest World Health Organization (WHO) statistics, 99% of the world’s population is living in places where air pollution levels exceed the WHO guideline limits, with 4.2 million premature deaths estimated worldwide [1]. In Europe, despite ongoing air quality improvements, air pollution is the greatest environmental health risk, causing cardiovascular and respiratory diseases, and preventable deaths [2]. Here, in 2021, 97% (76%) of the urban population was exposed to concentrations of PM_2.5_ (PM_10_) above the WHO limits [2]. The Padana Plain (Northern Italy) is one of the most polluted areas in Europe, particularly in terms of PM concentrations [3]. In 2022, PM_10_ concentrations in the Padana Plain were second only to those in the Balkan countries and in some industrial sites in Spain [4]. The number of annual exceedances of the PM_10_ daily limit value (50 μg/m^3^), stated by the EU Directive 2008/50/EC, reached up to 108 days, only being surpassed by that in the former Yugoslavia countries. In 2022, an average of 39.4 annual exceedances per station was recorded in the Padana Plain [4], which is higher than the maximum permitted value of 35. In Italy, air quality monitoring is officially regulated by the environmental protection agencies of each region (ARPAs), which measure PM_2.5_ and PM_10_ concentrations at daily resolution. This results in a significant knowledge gap on particulate matter (PM) temporal dynamics occurring during the day, which is critical if considering the severe PM conditions affecting the Padana Plain, particularly during the winter months [5]. This knowledge gap can be effectively filled by using low-cost (LC) sensors.

LC sensor technology has shown dramatic advances in air quality monitoring since the very first prototype developed by Bart et al. [6]. LC air pollution sensors have exhibited across the years an increasing ability to collect accurate, real-time, high spatial and temporal resolution measurements, which has favoured their development worldwide [7]. LC air quality networks are able to detect information on air pollution, temporal trends, hotspots and possibly source apportionment, that traditional (sparser) networks are unable to detect [8]. This has led to a change in air quality monitoring paradigm, as it is no longer exclusive to government organizations [9]. Current air quality legislation, on the other hand, is very inclusive towards LC sensors. In Europe, for example, the EU Directive 2008/50/EC officially incorporates LC monitors, assigning them a different regime than reference monitors, so that a lower accuracy is required to their measurements (called “indicative” rather than “regulatory”). Whereas in measuring PM concentrations, regulatory instruments typically use the gravimetric method, and LC sensors use indirect optical methods based on light scattering by particles [10]. Nephelometers and optical particle counters are the most commonly used LC sensors. The latter directly count particles according to their size, while nephelometers estimate particle density and convert it into particle mass [11]. In any case, the light-scattering approach is sensitive to variations in particle properties such as size distribution, shape and composition [12]. Increases in ambient humidity can affect hygroscopic growth of particles, and thus their light-scattering coefficient, so that the mass reported by optical sensors may be biased [13]. Therefore, the need to perform field calibration/validation of LC sensors by comparing their measurements to those from co-located regulatory stations is recommended when dealing with LC air sensor applications [7].

Over the last few years, PM measurements from several LC monitoring networks worldwide were made publicly available [14]. These networks provide geographic coverage extending from country-(e.g., Village Green, OpenSense) to continent (e.g., Citi-Sense, Smartcitizen) up to a global level (e.g., AirVisual, PurpleAir) [9]. Since most of such LC sensors only rely on calibration performed at manufacturer level (factory calibration), their pre/post-deployment evaluation vs. regulatory monitors is imperative [15]. Several worldwide performance evaluations of PM monitoring LC sensors under different real-world field conditions were performed over the years (e.g., [16]). Typically, LC units were tested vs. co-located (or at most, 1 km away) at reference stations, whereas in many practical applications, they were deployed in areas without a nearby reference system [17]. This is a serious issue, particularly in heavily PM-polluted areas, where it is instrumental to know the spatial representativeness of LC measurements and related citizen exposure.

The present study aims to bridge this gap by assessing the performance of LC sensors in monitoring PM_2.5_ and PM_10_ concentrations over a crucial hotspot such as the Padana Plain. LC sensors were assessed against various ARPA reference stations located nearby. Using reference PM_2.5_ and PM_10_ daily concentrations during the “heating season” that concurs with the most acute PM pollution episodes, two LC monitoring networks have been assessed and compared: (i) the well-established PurpleAir (https://www.purpleair.com, accessed on 16 April 2024); (ii) the newly emerging AirQino (https://www.airqino.it/en, accessed on 16 April 2024). The meaningfulness of this comparison is enhanced by the fact that these two networks are equipped with sensors built by different manufacturers. Finally, the advantages and the limitations of using LC sensor network data to improve the spatial and temporal scale of reference stations are discussed.

## 2. Materials and Methods

### 2.1. Study Area

**Figure 1:**
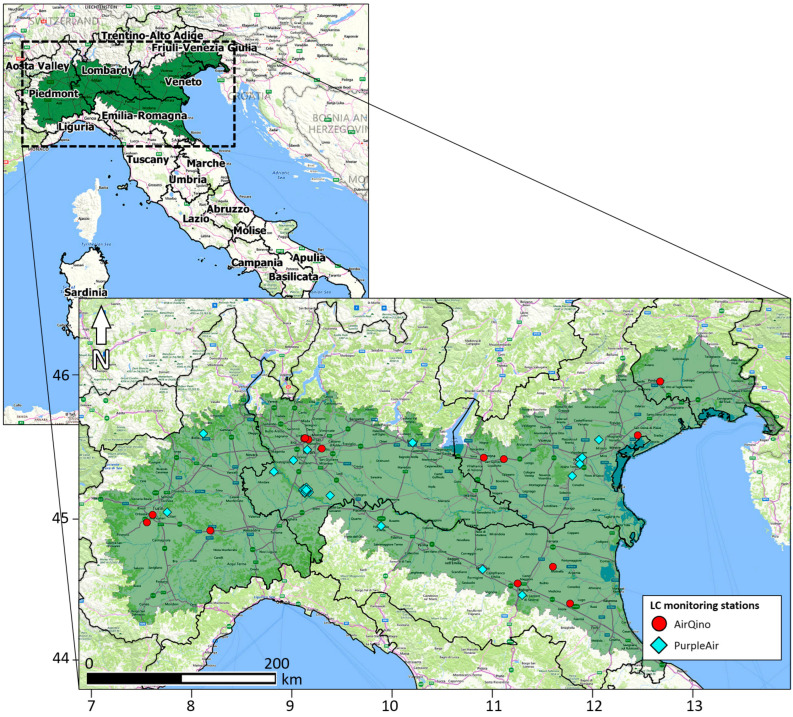
Map of the Padana Plain (green shaded area) in Italy, also showing the full operating LC PM_2.5_ and PM_10_ monitoring networks: AirQino (red dots); PurpleAir (cyan diamonds). Cartography basemap: Bing.

Located in northern Italy, the Padana Plain is a morphologically and hydrographically unitary region, largely made up by the Po river valley, and limited to the N by the Alps and to the S by the Northern Apennines (Figure 1).

The Padana Plain includes five regions: Piedmont, Lombardy, Emilia-Romagna, Veneto, and Friuli-Venezia Giulia. Providing an overall share of the gross domestic product (GDP) of 50.8% [18], the Padana Plain represents a fundamental economical asset to the country. With a surface area of 49,835 m^2^ (16.5% of the national territory), it is home to 19,208 million inhabitants, approximately one third (32.6%) of the entire countrywide population [19]. The urbanized surface area (6885 km^2^) is equal to 33.9% of the national one, while population density (385.43 inhab./m^2^) is almost double the Italian average.

The Padana Plain is a macro-region characterized by strong (often 2 or more times higher than national average) rates of the following: (i) anthropization; (ii) industrialization; (iii) road traffic; (iv) use of biomass heating systems; (v) agricultural and livestock activities. All these drivers result in large emissions of both primary PM and secondary PM precursors such as NO_x_ and NH_3_. The area is also characterized by strongly adverse meteorological conditions [20]. Particularly during colder months, the Alps and Apennines chains surrounding the region act as natural obstacles to winds and convective motions. This results in frequent wind calms and thermal inversion both at nighttime and daytime that cause the buildup and ageing of the intense emissions affecting the area [21].

### 2.2. Data

#### 2.2.1. PurpleAir Low-Cost Sensors

PurpleAir (https://www.purpleair.com, accessed on 16 April 2024) is an LC sensor-based PM monitoring network started in 2015 [22], which to date has more than 10,000 monitors deployed worldwide [23]. PurpleAir is based on the PA-II unit, which contains two Plantower PMS5003 sensors (Plantower, Beijing, China) providing real-time PM measurements, units’ inner pressure, temperature, humidity and a microcontroller to communicate with the two PMS5003 sensors and with the PurpleAir server [17]. The two PMS5003 sensors provide two sets of PM readings: (i) particle number, and (ii) mass concentration [14]. These sensors are based on the light-scattering principle, with a photodiode detector converting the scattered light to a voltage pulse. The number of pulses is then converted to the number of particles in sizes of 0.3, 0.5, 1.0, 2.5, 5.0 and 10 μm [17]. A complex mass conversion algorithm—not available to the public—is applied to convert the particle counts to PM_1.0_, PM_2.5_ and PM_10_ mass concentrations [23]. Noteworthy, Plantower PMS5003 sensors are factory calibrated using ambient aerosol across several cities in China [13].

Over the last few years, several studies have assessed the performance of PurpleAir PA-II sensors, particularly in the US, such as in Utah [24], Pennsylvania [13,25], Colorado [10,17], Washington [14] and California [22,23,26]. Further works have also been carried out in Canada [12], China [27], Greece [28] and Africa [29]. 

**Figure 2:**
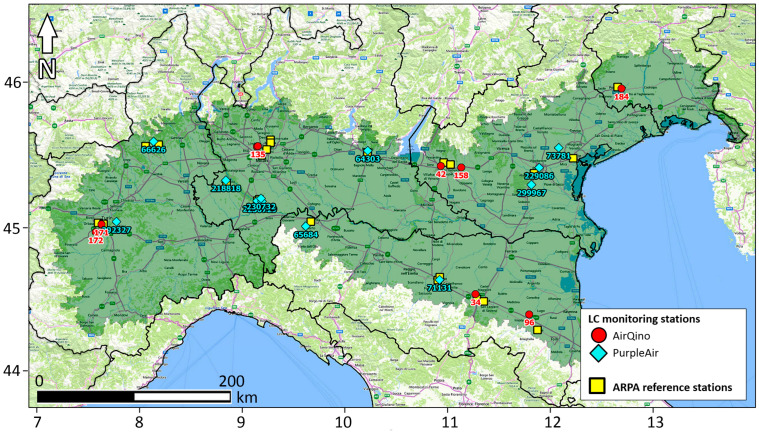
Map of PM_2.5_ and PM_10_ monitoring stations analyzed in the study: AirQino LC sensors (red dots); PurpleAir LC sensors (cyan diamonds); ARPA reference stations (yellow squares). Cartography basemap: Bing.

In the present study, PM_2.5_ and PM_10_ observations from PurpleAir PA-II sensors have been downloaded from OpenAQ (https://openaq.org, accessed on 16 April 2024). OpenAQ is an open-source platform that integrates air quality data publicly released by ground-based monitors around the world as divided into reference-grade stations and LC sensors [30]. Overall, PurpleAir includes 32 LC PM_2.5_ and PM_10_ monitoring stations in the Padana Plain (Figure 1). A subset of these PurpleAir stations have been used in the present study; their location is shown in Figure 2, while their characteristics are presented in Table 1.

#### 2.2.2. AirQino Low-Cost Sensors

AirQino (https://www.airqino.it/en, accessed on 16 April 2024) is an LC sensor-based air quality monitoring platform developed by CNR IBE [31]. The unit was conceived, created and implemented within national and international smart cities projects and device development started in 2014 [32,33]. The AirQino network currently consists of about 400 monitors mainly deployed in North and Central Italy (https://map.airqino.it, accessed on 16 April 2024). The unit is equipped with a modular set of industrial LC and high-resolution sensors aimed to collect gaseous pollutants (CO, NO_2_, O_3_), particulate matter (PM_2.5_ and PM_10_), CO_2_ and environmental parameters (noise, air temperature and relative humidity). The PM_2.5_ and PM_10_ sensors are based on the Novasense SDS011 detector (Nova Fitness, Jinan, China), based on the laser-scattering principle. The AirQino waterproof case was designed to minimize interference with reactive gases such as NO_2_ and O_3_; a small brushless fan creates a depression inside the box that attracts air from the inlet window. All sensors are placed beside the inlet window to reduce the contamination of fresh air inside the box (Figure S1). A microcontroller was designed and integrated into the sensor board to acquire, analyze and transmit all data through a GPRS 475G modem unit. Each device transmits geolocalized data to a central web server collecting records at high temporal and spatial resolution. Through a GIS engine and a web application, the data can be displayed, queried and analyzed in real time. The web platform integrates an SQL database and a web interface, while several APIs were developed to download data, either raw or calibrated, in Json or .csv format.

As detailed in the Supplementary Material of [34], the AirQino-integrated PM sensors were subject to various calibration and validation processes in recent years in central Italy [35,36]. The units were also tested in opposite extreme environments such as the Arctic region [37] and the Sub-Saharan Africa [34], thus proving capable of covering a full spectrum of climatic conditions and PM concentrations. Overall, a total of 14 LC PM_2.5_ and PM_10_ monitoring stations are operating in the Padana Plain (Figure 1). The location of AirQino stations used in the present study is shown in Figure 2, while their characteristics are reported in Table 1.

#### 2.2.3. ARPA Reference Stations

PM_2.5_ and PM_10_ reference observations have been retrieved from the ARPAs operating for each region. Data have been directly downloaded from the following websites (all accessed on 16 April 2024): https://aria.ambiente.piemonte.it/qualita-aria/dati (Piedmont region); https://www.arpalombardia.it/temi-ambientali/aria/form-richiesta-dati-stazioni-fisse (Lombardy); https://www.arpa.fvg.it/temi/temi/aria/sezioni-principali/download-indicatori-e-dati-aria/indicatori-giornalieri-qualita-aria/ (Friuli Venezia Giulia); https://sdati-test.datamb.it/arex (Emilia-Romagna). As for the Veneto region, PM data have been received upon request from local ARPA. PM monitoring was performed by each regional ARPA using a dual-channel filter-based gravimetric sampling method. The location of the ARPA stations used for assessing the LC stations is shown in Figure 2. Their detailed characteristics, along with the pairing with each LC station, are presented in Table S1 (PM_2.5_) and Table S2 (PM_10_ concentrations).

### 2.3. Methods

PM_2.5_ and PM_10_ concentrations were collected at hourly resolution by the LC stations and then averaged to daily values to match the resolution of ARPA data. Pairing of LC stations with ARPA stations was based on the following criteria: (i) availability of PM_2.5_ and/or PM_10_ data; (ii) geographic proximity (at most within 15 km); (iii) similarity in type; (iv) a minimum of 50% concurrently available data. This resulted in the LC vs. ARPA pairing reported in Table 1 and detailed in Table S1 (PM_2.5_) and Table S2 (PM_10_ sensors). To ensure robustness of the assessment process, the monitoring campaign—consistently with [38]—focused on the colder months, i.e., the “heating season” when biomass and wood combustion for heating contributed as an additional emission source to PM concentrations. According to the Italian regulations (DPR no. 412 of 26 August 1993), in the Padana Plain, a maximum 14 h of heating usage is allowed per day during the period from 15 October to 15 April Therefore, the 15 October 2022–15 April 2023 period was selected for the current study. It was also chosen because the climatic conditions in the region trigger the maximum PM levels. An outlier removal procedure based on the interquartile (IQR) range method was applied to all datasets, i.e., removing values below (above) the first (third) quartile minus (plus) 1.5 times the IQR range.

A descriptive statistical analysis of LC and ARPA time series, including 95% confidence interval of the mean, was performed. The following statistical scores were used to assess the LC observations: mean bias (MB), linear regression’s slope and intercept, mean absolute error (MAE), root mean squared error (RMSE), coefficient of determination (R^2^) and correlation coefficient (r). An F test was also performed to calculate the significance level of LC observations variance with respect to ARPA observations. To evaluate performance of LC sensors in monitoring PM_2.5_ daily concentrations vs. regulatory monitors, the target values recommended by the United States Environmental Protection Agency (US EPA) for the ‘‘base’’ testing [39] were considered. Indeed, comparison with these target values was intended as indicative rather than stringent. Since the regulatory stations were not co-located, a field assessment—rather than a proper field validation—was performed in the study. The target values for PM_2.5_ sensors are detailed in Table S3.

The “R-stat” environment vs. 4.3.1 [40] was used to perform all computations. The following packages integrated in “R-stat” (and functions implemented therein) were used: “pastecs” [41] for the descriptive statistical analysis (function “stat.desc”); “Metrics” [42] to calculate the statistical scores (functions “bias”, “mae”, “rmse”); the R Stats Package [43] to calculate other metrics (“sd”, “cor”), the linear regression (“lm”) and F test (“var.test”); the R Graphics Package [44] to plot boxplots (“boxplot”) and time series (“plot”); “ggplot2” [45] to draw the scatter plots (“ggplot”); “OpenAir” [46] to plot the Taylor diagrams [47] (“TaylorDiagram”). Other functions included in the R Base Package such as “mean”, “summary” and “apply” were used.

## 3. Results

### 3.1. PM_2.5_ and PM_10_ Observed Concentrations

Depending on availability of ARPA stations complying with the pairing criteria set in Section 2.3, a total of 19 LC stations (9 AirQino and 10 PurpleAir) were assessed for PM_2.5_ monitoring, while 20 LC stations (8 AirQino and 12 PurpleAir) were assessed for PM_10_ monitoring. Overall, ARPA stations were located at an average distance of about 6 km from LC stations, in the range of 1.340 ÷ 13.500 km for PM_2.5_ concentrations (Table S1), and 0.330 ÷ 13.650 km for PM_10_ concentrations (Table S2). The basic statistics of PM_2.5_ and PM_10_ daily concentrations measured by all LC stations compared to the corresponding ARPA stations are summarized, respectively, in Table 2 and Table 3.

ARPA reference stations paired to the AirQino stations return a lower overall mean value of PM_2.5_ daily concentrations than those paired to the PurpleAir stations (22.5 vs. 26.4 μg/m^3^, Table 2), thus being located in environments affected by lower pollution levels. AirQino stations overall under-estimate ARPA PM_2.5_ observations (17.8 vs. 22.5 μg/m^3^), while PurpleAir stations over-estimate them (30.5 vs. 26.4 μg/m^3^). Conversely, both AirQino and PurpleAir stations overall under-estimate ARPA PM_10_ daily concentrations (31.2 vs. 34.3 μg/m^3^ the former, 34.5 vs. 36.8 μg/m^3^ the latter, Table 3).

In Table S4, the statistics of PM_2.5_/PM_10_ daily concentration ratios measured by the LC and ARPA stations are also reported. ARPA stations paired to the AirQino stations lie in areas affected by a lower amount of fine particles than those paired to the PurpleAir stations (mean ratio of 0.67 vs. 0.72). The ARPA PM_2.5_/PM_10_ ratio is generally under-estimated by the former (0.57 vs. 0.67), while over-estimated by the latter (0.85 vs. 0.72).

**Figure 3:**
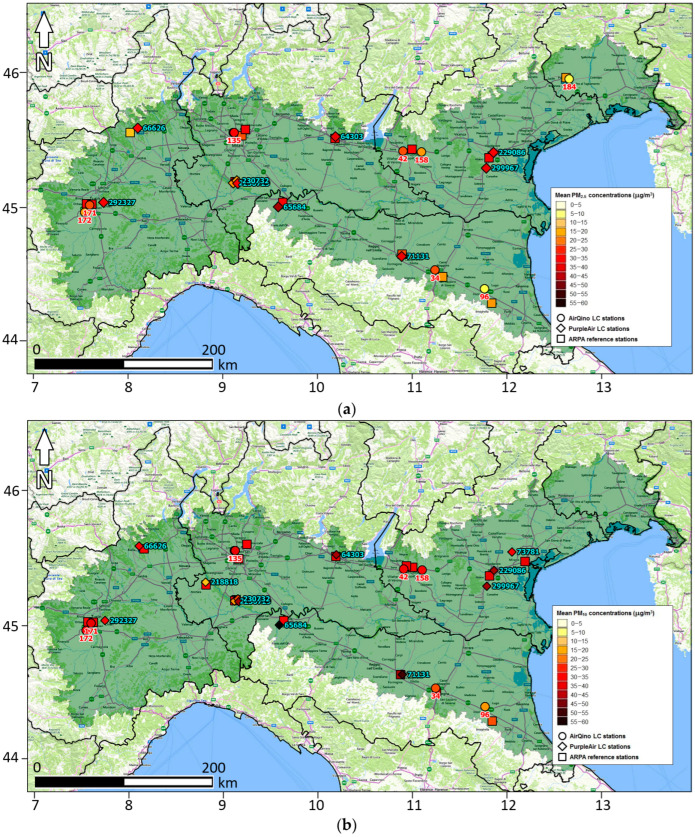
Map of full period averaged daily concentrations of (**a**) PM_2.5_ and (**b**) PM_10_ measured by LC stations and corresponding paired ARPA reference stations (15 October 2022–15 April 2023).

Figure 3 shows the map of PM_2.5_ and PM_10_ daily concentrations averaged over the full period at each LC station as compared to the corresponding ARPA stations.

AirQino stations reproduce ARPA PM_2.5_ observations quite well (Figure 3a), with the highest discrepancies (percentage under-estimations at worst between 40 and 60%) occurring in the eastern part of the study area (station IDs 96 and 184). PurpleAir stations are slightly finer overall than AirQino stations, though locally exhibiting higher discrepancies (percentage over-estimations at worst between 60 and 100%) occurring inland throughout the domain (IDs 65684, 66626 and 71131).

AirQino stations are particularly reliable in monitoring PM_10_ daily concentrations (Figure 3b); they systematically under-estimate reference observations, though exhibiting a percentage bias of, at worst, 28% (again at ID = 96). Conversely, PurpleAir stations exhibit both significant over-estimations (at most 50%, ID = 65684) and under-estimations (at most 40–60%, IDs 230713, 230732 and 218818).

### 3.2. Scores by Station of Low-Cost Stations

The statistical scores exhibited by each LC station against the reference stations are detailed in Table 4 (PM_2.5_) and Table 5 (PM_10_ concentrations). The scores in monitoring the corresponding PM_2.5_/PM_10_ daily concentration ratios are reported in Table S5.

LC sensors were assessed in meeting the target values of US EPA “base” testing for PM_2.5_ air sensors (Table S3). Both the linear regression’s slope and intercept are concurrently met by four (out of nine) AirQino sensors, and by two (out of ten) PurpleAir sensors (Table 4). The linearity attribute (R^2^ ≥ 0.70) is satisfied by three AirQino and eight PurpleAir PM_2.5_ sensors. All LC sensors (except AirQino ID 184) lack precision, as the standard deviation of PM_2.5_ measured concentrations is above 5 μg/m^3^ (Table 2). Also, the attribute of error is systematically failed, as all LC sensors exhibit RMSE values above 7 μg/m^3^ (Table 4). 

**Figure 4:**
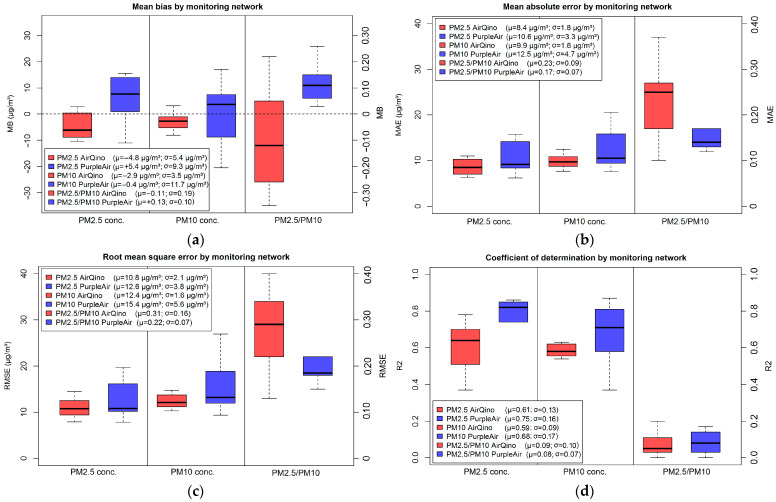
Boxplots of statistical skill scores of LC stations vs. ARPA reference stations in monitoring daily concentrations of PM_2.5_, PM_10_ and PM_2.5_/PM_10_ ratio (15 October 2022–15 April 2023): (**a**) mean bias; (**b**) mean absolute error; (**c**) root mean square error; (**d**) coefficient of determination. Boxplots are delimited by the first (*Q*_1_) and third (*Q*_3_) distribution’s quartiles, while the black line inside the box denotes the median value (*Q*_2_). The mean and standard deviation values by pollutant for each LC network are reported in brackets.

The boxplots of MB, MAE, RMSE and R^2^ distributions, exhibited by all LC stations as detailed in Table 4, Table 5 and Table S5, are plotted in Figure 4.

The analysis of MB boxplots (Figure 4a) reveals that AirQino stations tend to under-estimate both PM_2.5_ and PM_10_ concentrations observed by the reference stations, at worst under-estimating the former by 10.4 and the latter by 8.1 μg/m^3^ (Table 4). Conversely, PurpleAir stations both under- and over-estimate ARPA observations, also exhibiting a wider MB distribution—and thus higher MB extreme values—than AirQino stations (Figure 4a). AirQino MB scores are affected by a lower spread than PurpleAir, markedly in monitoring PM_10_ concentrations (σ = 3.5 vs. 11.7 μg/m^3^). In both MAE (Figure 4b) and RMSE (Figure 4c) boxplots and for both pollutants, AirQino stations exhibit better scores than PurpleAir stations, not only if considering the lower mean values (MAE = 8.4 ÷ 9.9 and RMSE = 10.8 ÷ 12.4 μg/m^3^), but also the narrower distributions’ full range. Conversely, PurpleAir outplays AirQino in R^2^ values related to both PM_2.5_ and PM_10_ daily concentrations (Figure 4d). If considering the full range of R^2^ distribution, in measuring PM_2.5_ concentrations, R^2^ spans 0.37 ÷ 0.78 for AirQino and 0.41 ÷ 0.86 for PurpleAir stations, while for PM_10_ concentrations, R^2^ spans 0.42 ÷ 0.76 (AirQino) and 0.37 ÷ 0.87 (PurpleAir, Table 4).

Both LC networks fail to reproduce magnitude (Figure 4a) and time variation (Figure 4d) of the PM_2.5_/PM_10_ ratio. In terms of MAE (Figure 4b) and RMSE (Figure 4c), PurpleAir stations are less inaccurate than AirQino stations.

**Figure 5:**
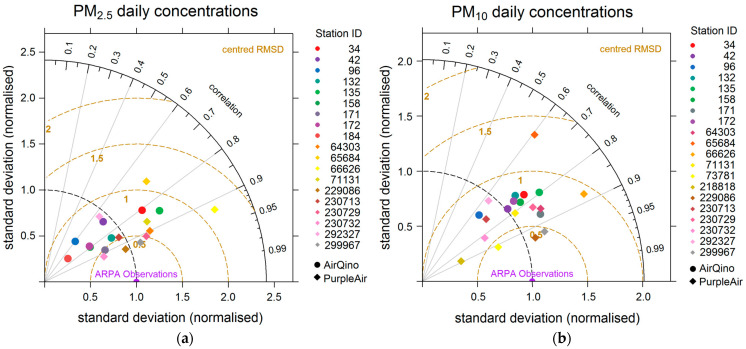
Taylor diagrams of (**a**) PM_2.5_ and (**b**) PM_10_ daily concentrations observed by all single LC stations compared to the paired ARPA reference stations (15 October 2022–15 April 2023). Dashed black circles (radial distance from the origin) show standard deviation of LC observations (σ_LC_) normalized to standard deviation of ARPA observations (σ_ARPA_). Concentric dashed yellow circles emanating from ARPA observations point show a centred root mean square difference (RMSD), which is also normalized to σ_ARPA_. RMSD is centred as mean values of LC and ARPA observations are subtracted first: therefore, the diagram does not provide information about overall biases [47].

The analysis of Taylor diagram provides further insight into LC station performances, markedly focusing on each individual station (Figure 5).

Several AirQino stations show a lower variability than ARPA reference stations (σ_LC_ < σ_ARPA_) in monitoring PM_2.5_ daily concentrations (Figure 5a), while PurpleAir stations exhibit a variability comparable or moderately higher than ARPA stations. PurpleAir stations show a lower centred RMSD and higher correlation coefficients than AirQino stations, with some points particularly close to the “ARPA observations” optimal point. In monitoring PM_10_ daily concentrations (Figure 5b), as shown by the cloud of points very close to each other, AirQino stations exhibit quite similar behaviour in all the three metrics of the Taylor diagram. Conversely, the PurpleAir station points show a large scatter, particularly in terms of variability with respect to ARPA variability. Also, in measuring PM_10_ daily concentrations, PurpleAir stations return a lower RMSD and higher correlation coefficients than AirQino stations.

### 3.3. Overall Scores by Monitoring Network of Low-Cost Stations

**Figure 6:**
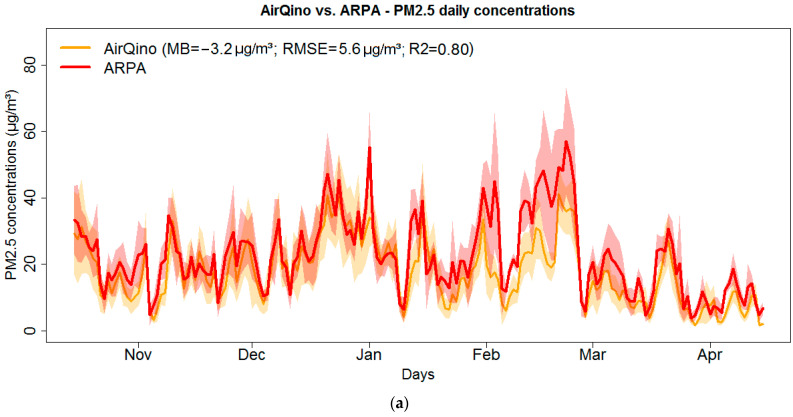

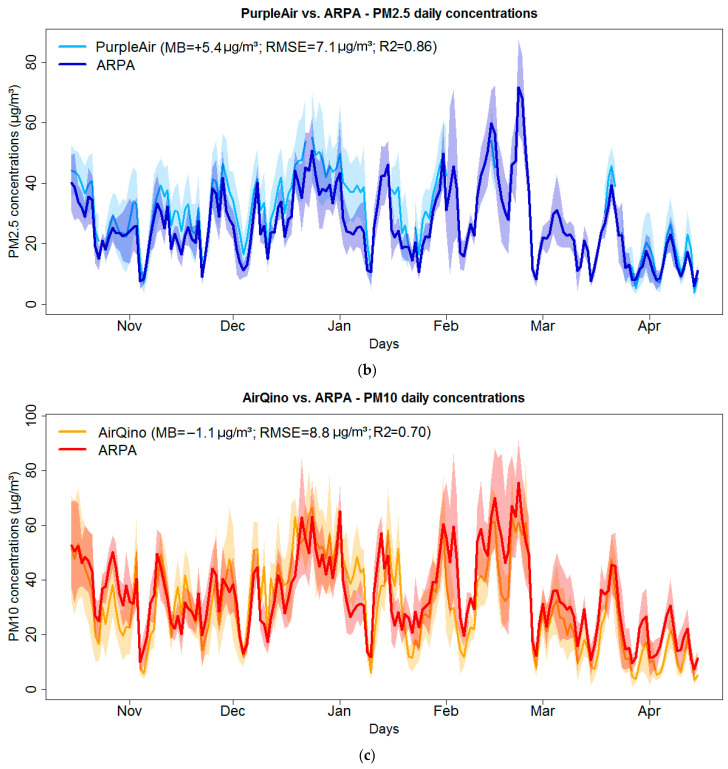

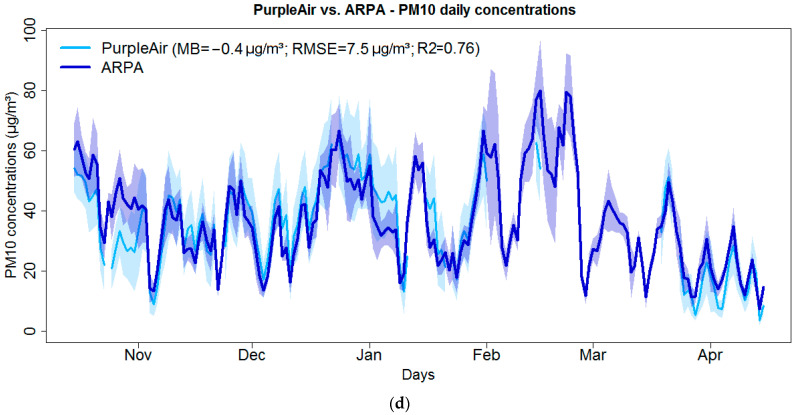
Time series of daily concentrations averaged by LC monitoring network compared to corresponding ARPA reference stations (15 October 2022–15 April 2023): (**a**) AirQino PM_2.5_; (**b**) PurpleAir PM_2.5_; (**c**) AirQino PM_10_; (**d**) PurpleAir PM_10_. For each LC monitoring network, mean bias, root mean square error and coefficient of determination are also shown. The shaded areas show the confidence interval at 95% level around the mean values.

Full time series of PM_2.5_ and PM_10_ daily concentrations averaged day-by-day by LC monitoring network vs. corresponding ARPA stations are plotted in Figure 6.

AirQino stations quite reasonably capture the pattern of ARPA PM_2.5_ concentrations, both in magnitude and time variation (RMSE = 5.6 μg/m^3^ and R^2^ = 0.80, Figure 6a). A closer analysis, however, reveals that AirQino measurements were particularly fine until late January and from March on, while they generally fail to reproduce ARPA observations during the month of February, i.e., when the highest ARPA concentrations are recorded. PurpleAir stations prove to remarkably capture the time variation of PM_2.5_ ARPA observations (R^2^ = 0.86, Figure 6b). Compared to AirQino stations, they exhibit better R^2^ values, while they exhibit worse values of MB (+5.4 μg/m^3^) and RMSE (7.1 μg/m^3^). However, it should be noted that PurpleAir PM_2.5_ observations were not available for comparison from early February to mid-March, which might possibly account for their overall better correlation than AirQino stations (R^2^ = 0.86 vs. 0.80). Compared to the case of PM_2.5_ concentrations, in monitoring PM_10_ concentrations, AirQino stations better fit the magnitude of reference observations, as returning an overall mean under-estimation of 1.1 μg/m^3^ (Figure 6c), while time variation is lower (R^2^ = 0.70). In this case, the February critical period appears to be better captured. Also, when monitoring PM_10_ concentrations (Figure 6d), PurpleAir stations better fit the magnitude (0.4 μg/m^3^ under-estimation) and time variation (R^2^ = 0.76) of reference observations with respect to AirQino stations, although, again, their missing values from early February to mid-March might affect this outcome.

**Figure 7:**
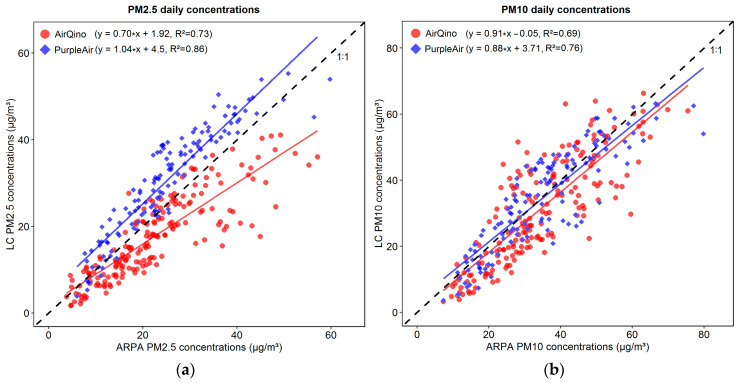
Scatter plots between daily concentrations averaged by LC monitoring network and corresponding ARPA reference stations (15 October 2022–15 April 2023): (**a**) PM_2.5_; (**b**) PM_10_. The best linear fit for each LC network, along with the corresponding equation (y = a * x + b, in brackets), are also shown as compared to the 1:1 black dashed line.

Figure 7 shows the scatter plot between the PM_2.5_ and PM_10_ daily concentrations averaged day-by-day by LC monitoring network vs. corresponding ARPA stations.

Figure 7a confirms PurpleAir higher accuracy than AirQino in monitoring ARPA PM_2.5_ daily concentrations (R^2^ = 0.86 vs. 0.73). The slope of PurpleAir linear best fit basically matches that of the perfect agreement (1:1) line. Therefore, PurpleAir well reproduces ARPA PM_2.5_ daily observations over their full range, apart from a positive offset factor (4.5 μg/m^3^) indicating a systematic over-estimation. Conversely, AirQino tends to under-estimate PM_2.5_ reference observations, particularly at the highest values.

The pattern of the two LC networks is quite similar when focusing on PM_10_ concentrations, as highlighted by their almost matching best-fit lines (Figure 7b). Compared to ARPA observations, PurpleAir measurements slightly over-estimate at lower concentrations and slightly under-estimate at higher concentrations, while AirQino measurements slightly under-estimate almost across the full range of concentrations. Again, PurpleAir shows higher skills than AirQino in fitting the reference observations (R^2^ = 0.76 vs. 0.69).

## 4. Discussion

PM_2.5_ daily concentrations observed in the Padana Plain by the ARPA reference stations are in better agreement with the newly emerging AirQino sensors than the well-established PurpleAir sensors, with better values of MB, RMSE and MAE (Figure 4a–c). As for PM_10_ daily concentrations, PurpleAir sensors are more accurate in fitting the magnitude (Figure 4a), while less accurate in terms of RMSE and MAE (Figure 4b,c). This lower magnitude of the error exhibited by AirQino vs. PurpleAir stations might be due to the fact that their paired reference stations are located in environments affected by lower PM_2.5_ (Table 3) and PM_10_ concentrations (Table 4). By contrast, PurpleAir sensors are better at capturing the linearity of responses and time variation of both PM_2.5_ and PM_10_ daily concentrations, as shown with higher values of R^2^ (Figure 4d) and r (Figure 5). This confirms findings on sensor performance reported, e.g., by Badura et al. [48]. Furthermore, it might depend on the average distance of ARPA reference stations from PurpleAir stations, which is lower than that from AirQino stations for both PM_2.5_ (4.6 vs. 7.5 km, Table S1) and PM_10_ concentrations (4.8 vs. 9.0 km, Table S2). Current PurpleAir PM_2.5_ performance is consistent with that reported by Ardon-Dryer et al. [17] within a 2-year (January 2017 to December 2018) similar field assessment (no co-location) of 46 units vs. (gravimetric-based) regulatory stations in four US cities. As in Figure 4a, Figure 6b and Figure 7a, the majority of PurpleAir units measured PM_2.5_ concentrations higher than the reference stations. As suggested by Ardon-Dryer et al. [17], this could likely be due to changes in relative humidity, whose values are particularly high in winter in the Padana Plain.

Although a rigorous field validation (co-location vs. regulatory stations) was not performed in the present study, it is insightful to compare current LC sensor scores with those resulting from field validation studies addressed in the literature. Due to this reason, the present scores deserve more credit. Within a 4-month (September to December 2020) field validation of three PurpleAir units vs. a co-located (gravimetric-based) regulatory station in Vancouver (Canada), in monitoring PM_2.5_ daily concentrations Zimmerman [12] reported better scores (RMSE = 7.64–10.13 μg/m^3^, R^2^ = 0.91–0.94) than those achieved in the Padana Plain (RMSE = 7.9–19.7 μg/m^3^, R^2^ = 0.41–0.86, Table 4). PurpleAir tendency of over-estimating PM_2.5_ observed concentrations (Figure 4a and Figure 6b) is consistent with the findings by Zimmerman [12] and Barkjohn et al. [49], who reported that PurpleAir sensors over-estimated PM_2.5_ concentrations by about 40% in most parts of the US. Current PurpleAir R^2^ values in monitoring PM_2.5_ daily concentrations (Table 4 and Figure 4d) are worse than those (R^2^ > 0.88) achieved by Sayahi et al. [24] in Salt Lake City (USA), where they co-located for 320 days 2 PurpleAir units vs. a reference (gravimetric-based) station. Compared to the R^2^ values resulting from a 39-day (December 2016 to January 2017) field validation of three PurpleAir units vs. two (optical-based) regulatory stations in California (USA) [50], current results are worse for PM_2.5_ (R^2^ = 0.41–0.86 vs. 0.93–0.97, Table 4), while they are comparable for PM_10_ daily concentrations (R^2^ = 0.37–0.87 vs. 0.66–0.70, Table 5). Agreeing with findings in [50], in the Padana Plain, PurpleAir sensors are confirmed to be more accurate in monitoring PM_2.5_ than PM_10_ daily concentrations (Figure 4d). After deploying an AirQino unit close to an ARPA reference station in Florence (Italy) across a full “heating season” (November 2016 to April 2017), Cavaliere et al. [35] returned scores in measuring both PM_2.5_ (MB = +4.39 μg/m^3^, RMSE = 7.95 μg/m^3^ and R^2^ = 0.90) and PM_10_ daily concentrations (MB = +0.72 μg/m^3^, RMSE = 7.80 μg/m^3^ and R^2^ = 0.84) better than those achieved in the Padana Plain (Table 4 and Table 5). Within a 78-day co-location vs. an ARPA station in Capannori (Italy) across a spring period in 2019, Brilli et al. [36] reported AirQino scores in measuring PM_2.5_ daily concentrations to be both better (RMSE = 4.2 μg/m^3^ vs. 7.9–14.6 μg/m^3^) and worse (R^2^ = 0.54 vs. 0.37–0.78) than those achieved in the present study (Table 4). The AirQino scores reported in the same study in monitoring PM_10_ daily concentrations were better in terms of RMSE (4.6 μg/m^3^) and comparable in terms of R^2^ (0.63, Table 5).

The performance of the single sensors mounted on both PurpleAir and AirQino devices was also analyzed. Coker et al. [51] co-located for one year (January to December 2020) one Plantower MS5003 sensor to a reference (gravimetric-based) monitor installed at the US embassy in Kampala (Uganda). They reported scores in monitoring PM_2.5_ daily concentrations (RMSE = 14.43 μg/m^3^ and R^2^ = 0.76) quite in line with those achieved by the same sensors mounted on the PurpleAir units in the Padana Plain (Table 4). Three Novasense SDS011 sensors were tested by Božilov et al. [52] against a (gravimetric-based) reference monitor at two cities in Serbia for two 2-week heating periods between 2021 and 2022. In measuring PM_2.5_ concentrations, they found the RMSE to be ranging between 3.8 ÷ 22.4 μg/m^3^ and R^2^ 0.55 ÷ 0.82, while in measuring PM_10_ concentrations, they reported the RMSE to be ranging between 11.1 ÷ 27.0 μg/m^3^ and R^2^ 0.52 ÷ 0.80. Compared to those achieved in the Padana Plain by the same sensors mounted on the AirQino units, these scores are better in terms of R^2^ and worse in terms of RMSE (Table 4). Better R^2^ scores (0.87–0.90) in measuring PM_2.5_ daily concentrations were also achieved by Badura et al. [48] while testing three Novasense SDS011 sensors vs. a co-located (gravimetric-based) reference monitor in Wrocław (Poland) between August 2017 and February 2018. 

The indicative compliance of current LC PM_2.5_ sensors to the US EPA “base” testing [39] was also explored. Assessed in meeting the corresponding target values (Table S3), the AirQino PM_2.5_ sensors show better bias and the PurpleAir PM_2.5_ sensors show better linearity (Table 4). Conversely, sensors belonging to both networks lack the attributes of precision (standard deviation above 5 μg/m^3^, Table 2) and error (RMSE above 7 μg/m^3^, Table 4).

If comparing LC sensor performance by pollutant, both PurpleAir and AirQino sensors exhibit higher accuracy in fitting the magnitude of PM_10_ than PM_2.5_ observations, while lower accuracy in terms of RMSE, MAE and R^2^ (Figure 4). This outcome is consistent with findings from the above studies for both PurpleAir [50] and AirQino sensors [35]. In monitoring PM_2.5_ concentrations, the PurpleAir sensors are stable across the full range of observations, unlike the AirQino sensors, whose under-estimations increase as the observations increase (Figure 7a). By contrast, in monitoring PM_10_ concentrations, the pattern of the two LC networks is similar (Figure 7b). This different behaviour per pollutant is noteworthy, particularly if considering that for both PurpleAir and AirQino sensors the PM_2.5_ and PM_10_ concentration outputs are produced after a mass conversion procedure based on a single input signal (Section 2.2.1 and Section 2.2.2). This is apparent in the PurpleAir sensors, whose PM_2.5_ outputs follow the 1:1 line (apart from an over-estimation offset factor, Figure 7a), while their PM_10_ outputs show a slight under-estimation trend (Figure 7b). Since Plantower PMS5003 sensors mounted on PurpleAir monitors are factory calibrated based on PM measurements collected in China [13], it can be recommended to introduce a correction factor to suitably reduce the estimated PM_2.5_/PM_10_ ratio. The latter (as shown in Figure 4a and in Table S5) is on average higher than the ARPA observed value. Likewise, since the Novasense SDS011 sensors mounted on AirQino monitors are factory calibrated based on China measurements as well, a correction should also be introduced to the AirQino outputs in order to increase (rather than decrease) their PM_2.5_/PM_10_ ratios. After these adjustments, the weight of fine fraction in total PM_10_ concentrations should be increased for AirQino and reduced for PurpleAir sensors. The boxplots of MAE (Figure 4b) and RMSE (Figure 4c) related to the PM_2.5_/PM_10_ ratios suggest that such corrections could be more successful for PurpleAir than AirQino sensors, since the former are affected by a lower discrepancy. In any case, the particularly low R^2^ values exhibited by both LC sensors (Figure 4d) suggest their inability at capturing the time variation of PM_2.5_/PM_10_ ratio. 

## 5. Conclusions

In recent years, public availability of increasingly extensive LC sensor-based PM monitoring networks worldwide allowed researchers and citizen scientists to take huge advantage from the LC sensor technology. However, it should be borne in mind that most such sensors are only factory calibrated and factory calibration procedures are not specified in their datasheets [48]. Thus, a proper on-site calibration should necessarily be performed prior to using their data [52]. Unfortunately, this calibration is often not viable as LC sensors are frequently placed in regions where reference systems nearby are missing [17]. In the present study, the performance of two LC monitoring networks in measuring PM_2.5_ and PM_10_ concentrations compared to reference stations over a crucial PM hotspot such as the Padana Plain (Italy) was analyzed. Both the well-established PurpleAir and the newly emerging AirQino sensors returned good performance. AirQino sensors were finer in fitting the magnitude of PM_2.5_ concentrations, while PurpleAir sensors were in fitting that of PM_10_ concentrations. PurpleAir sensors were better at capturing the linearity and time variation of both PM_2.5_ and PM_10_ concentrations. Conversely, findings from several studies (e.g., [9,12,13,49]) were confirmed about LC sensor difficulties in correctly discriminating the size of individual particles as a result of the light-scattering PM sampling method they are based upon.

Indeed, finding a suitable tuning of the PM_2.5_ vs. PM_10_ outputs falls into an ever-wider research line aimed at developing the best correction techniques for LC sensors. Several such studies for PurpleAir sensors have been carried out in the literature. However, as pointed out by Barkjohn et al. [49], corrections have been developed for a specific region [22,28], season [24], or atmospheric condition [17], while few studies have addressed how widely applicable they are. Incorporating environmental factors such as air temperature and particularly relative humidity proved to return the finest scores [12,17,28,49]. However, finding the best correction algorithm valid for all application conditions remains a very challenging and certainly open issue that needs further efforts. This is particularly urgent in highly PM-polluted areas such as the Padana Plain. Here, high-granularity monitoring networks based on sufficiently reliable LC sensors could give a new dimension to air quality monitoring and democratize the whole process by making monitoring and results directly accessible at the community level [23].

The main limitation of this study lies in that, since the regulatory stations were not close enough to allow co-location, it was not possible to perform a rigorous field validation of the LC sensors. In addition, since regulatory observations were sampled at a daily resolution, PM_2.5_ and PM_10_ concentrations collected by the LC stations were assessed at 24 h rather than their native 1 h resolution. Future research lines could include deployment of new AirQino stations in the Padana Plain—hopefully very close to the existing ARPA stations—as well as using current PM observations from the two LC networks for validating PM forecasting products in the Padana Plain such as, e.g., the Copernicus Atmosphere Monitoring Service (CAMS).

## Figures and Tables

**Table 1:** Characteristics of LC air quality stations sorted by monitoring network and ARPA reference stations available for assessment ^1^.

Network	ID	Type ^2^	Latitude (Deg N)	Longitude (Deg E)	Elevation (m a.s.l.)	Assessment vs. ARPA Stations ^3^	
						PM_2.5_	PM_10_
AirQino	34	RB	44.5336	11.2736	38	x	x
	42	SB	45.4220	10.9343	75	x	x
	96	RB	44.3929	11.7959	19	x	x
	132	SB	45.5562	9.1422	158	x	x
	135	RT	45.5575	9.1520	159	x	x
	158	RT	45.4129	11.1315	42	x	x
	171	UB	45.0194	7.6285	246	x	x
	172	RB	44.9651	7.5732	245	x	x
	184	SB	45.9523	12.6914	23	x	
PurpleAir	64303	UB	45.5271	10.2230	131	x	x
	65684	SB	45.0039	9.6196	86	x	x
	66626	RB	45.5902	8.1347	383	x	x
	71131	UT	44.6351	10.9208	36	x	x
	73781	SB	45.5482	12.0797	14		x
	218818	SB	45.3252	8.8417	112		x
	229086	UB	45.4095	11.8895	12	x	x
	230713	UT	45.1827	9.1460	65	x	x
	230729	RB	45.1803	9.1913	73	x	x
	230732	RB	45.1994	9.1814	81	x	x
	292327	SB	45.0375	7.7728	455	x	x
	299967	SB	45.2945	11.8166	7	x	x

^1^ Geographic location of LC air quality stations is presented in Figure 2. ^2^ Station type: UB Urban background; RB, Rural background; SB, Suburban background; UT, Urban traffic; RT, Rural traffic. ^3^ Assessment of PM_2.5_ and PM_10_ LC sensors is subject to availability of complying ARPA stations.

**Table 2:** Statistics of PM_2.5_ daily concentrations measured by LC stations and corresponding paired ARPA reference stations (15 October 2022–15 April 2023).

LC Stations						ARPA Stations	
Network	ID	Type ^1^	Valid Data (%) ^2^	PM_2.5_ Concentrations (µg/m^3^)		PM_2.5_ Concentrations (µg/m^3^)	
				Mean	St.dev. ^4^	Mean	St.dev.
AirQino	34	RB	92.4	20.2	14.1	17.6	10.7
	42	SB	75.4	21.2	13.7	20.8	15.0
	96	RB	60.7	9.1	5.5	15.2	9.9
	132	SB	91.8	18.7	11.3	26.8	13.0
	135	RT	92.9	25.6	16.3	22.8	11.1
	158	RT	78.7	16.3	11.5	25.1	18.5
	171	UB	91.3	20.1	9.0	25.5	12.1
	172	RB	89.6	15.0	7.2	25.2	11.5
	184	SB	65.6	6.0	**3.2**	16.4	9.0
	Overall ^3^			17.8	9.5	22.5	11.5
PurpleAir	64303	UB	65.0	35.3	16.3	28.4	12.8
	65684	SB	66.7	40.0	18.9	25.5	12.2
	66626	RB	65.0	27.8	14.1	13.8	7.0
	71131	UT	69.4	39.3	15.0	23.7	11.6
	229086	UB	66.7	33.7	16.4	29.2	17.2
	230713	UT	58.5	16.0	11.2	27.0	11.9
	230729	RB	61.2	34.5	14.9	26.2	12.3
	230732	RB	58.5	17.5	8.6	26.1	12.2
	292327	SB	66.1	27.6	12.0	26.6	13.0
	299967	SB	65.0	37.6	18.3	29.0	16.2
	Overall ^3^			30.5	12.1	26.4	12.1

^1^ Station type: UB, urban background; RB, rural background; SB, suburban background; UT, urban traffic; RT, rural traffic. ^2^ For each LC station and pollutant, valid data refer to the sample of concurrently available LC and ARPA observations. ^3^ Overall values by monitoring network of mean concentrations averaged across the full period are based on day-by-day values averaged across all LC or ARPA stations. ^4^ Values in bold for standard deviation denote LC stations meeting US EPA target values recommended for PM_2.5_ air sensors (Table S3).

**Table 3:** Statistics of PM_10_ daily concentrations measured by LC stations and corresponding paired ARPA reference stations (15 October 2022–15 April 2023).

LC Stations						ARPA Stations	
Network	ID	Type ^1^	Valid Data (%) ^2^	PM_10_ Concentrations (µg/m^3^)		PM_10_ Concentrations (µg/m^3^)	
				Mean	St.dev.	Mean	St.dev.
AirQino	34	RB	96.7	23.2	16.4	26.9	13.5
	42	SB	79.2	31.0	18.1	34.5	17.8
	96	RB	63.4	17.5	9.5	24.3	12.0
	132	SB	85.8	31.3	19.5	32.8	17.0
	135	RT	97.3	38.2	23.1	38.8	17.3
	158	RT	92.4	28.1	19.5	36.2	17.0
	171	UB	90.7	36.8	19.8	33.7	16.1
	172	RB	92.4	31.5	16.2	33.3	14.7
	Overall ^3^			31.2	15.9	34.3	14.5
PurpleAir	64303	UB	66.1	41.9	19.8	39.5	15.7
	65684	SB	67.8	51.0	26.3	33.9	15.7
	66626	RB	68.9	32.7	17.5	25.3	10.5
	71131	UT	70.5	47.7	19.7	41.2	18.9
	73781	SB	71.0	33.8	19.0	38.0	25.2
	218818	SB	62.3	12.4	7.0	33.0	17.6
	229086	UB	68.9	41.4	22.1	36.5	20.1
	230713	UT	66.7	17.6	13.7	35.8	17.0
	230729	RB	67.8	41.3	18.6	33.8	15.4
	230732	RB	65.0	20.3	10.5	33.7	15.2
	292327	SB	65.6	32.7	15.6	35.8	16.4
	299967	SB	68.3	44.4	22.9	35.9	19.1
	Overall ^3^			34.5	14.8	36.8	15.8

^1^ Station type: UB, urban background; RB, rural background; SB, suburban background; UT, urban traffic; RT, rural traffic. ^2^ For each LC station and pollutant, valid data refer to the sample of concurrently available LC and ARPA observations. ^3^ Overall values by monitoring network of mean concentrations averaged across the full period are based on day-by-day values averaged across all LC or ARPA stations.

**Table 4:** Statistical scores of LC stations compared to ARPA reference stations in measuring PM_2.5_ daily concentrations (15 October 2022–15 April 2023) ^1,2^.

Network	ID	Valid Data (%)	MB (µg/m^3^)	MAE (µg/m^3^)	Slope	Intercept (µg/m^3^)	RMSE (µg/m^3^)	R^2^
AirQino	34	92.4	+2.6	6.6	**1.06**	**1.5**	8.7	0.65
	42	75.4	+0.4	8.5	0.64	7.9	11.2	0.49
	96	60.7	−6.2	7.1	0.33	**4.0**	10.0	0.37
	132	91.8	−8.1	8.6	**0.73**	**−0.8**	10.8	**0.70**
	135	92.9	+2.8	7.0	**1.25**	**−2.9**	9.4	**0.72**
	158	78.7	−8.8	11.0	0.50	**3.8**	14.6	0.64
	171	91.3	−5.4	6.3	**0.66**	**3.4**	7.9	**0.78**
	172	89.6	−10.1	10.3	0.49	**2.7**	12.5	0.61
	184	65.6	−10.4	10.5	0.25	**1.8**	12.6	0.51
PurpleAir	64303	65.0	+7.0	8.4	**1.15**	**2.8**	10.1	**0.81**
	65684	66.7	+14.6	15.3	**1.11**	11.8	19.7	0.51
	66626	65.0	+14.0	14.2	1.85	**2.3**	16.2	**0.85**
	71131	69.4	+15.6	15.7	**1.12**	12.8	17.4	**0.74**
	229086	66.7	+4.5	6.2	**0.88**	8.0	7.9	**0.86**
	230713	58.5	−11.0	11.1	**0.81**	−5.9	12.7	**0.74**
	230729	61.2	+8.4	9.2	**1.11**	5.5	10.4	**0.83**
	230732	58.5	−8.6	8.6	**0.65**	**0.6**	10.2	**0.85**
	292327	66.1	+1.0	7.9	0.60	11.7	10.6	0.41
	299967	65.0	+8.6	9.1	**1.04**	7.3	11.0	**0.86**

^1^ All R^2^ scores significant at 1% level (*p* < 0.05). ^2^ Values in bold for slope, intercept, RMSE and R^2^ denote LC stations meeting US EPA target values recommended for PM_2.5_ air sensors (Table S3).

**Table 5:** Statistical scores of LC stations compared to ARPA reference stations in measuring PM_10_ daily concentrations (15 October 2022–15 April 2023) ^1^.

Network	ID	Valid Data (%)	MB (µg/m^3^)	MAE (µg/m^3^)	Slope	Intercept (µg/m^3^)	RMSE (µg/m^3^)	R^2^
AirQino	34	96.7	−3.8	9.2	0.92	−1.7	11.3	0.58
	42	79.2	−3.4	10.3	0.78	4.3	12.8	0.58
	96	63.4	−6.8	8.8	0.52	5.0	11.4	0.42
	132	85.8	−1.5	10.8	0.84	3.7	13.5	0.54
	135	97.3	−0.6	10.9	1.06	−3.0	14.0	0.63
	158	92.4	−8.1	12.5	0.89	−4.1	14.8	0.61
	171	90.7	+3.2	7.7	1.07	0.8	10.3	0.76
	172	92.4	−1.9	8.7	0.83	3.8	11.1	0.57
PurpleAir	64303	66.1	+2.4	9.1	1.07	−0.5	10.7	0.73
	65684	67.8	+17.1	20.4	1.02	16.4	26.9	0.37
	66626	68.9	+7.4	9.7	1.47	−4.4	12.2	0.77
	71131	70.5	+6.6	11.7	0.84	13.1	13.7	0.65
	73781	71.0	−4.2	8.1	0.69	7.6	11.8	0.83
	218818	62.3	−20.6	20.6	0.35	0.8	23.7	0.79
	229086	68.9	+5.0	7.6	1.03	4.0	9.4	0.87
	230713	66.7	−18.1	18.2	0.58	−3.1	21.7	0.51
	230729	67.8	+7.5	11.0	1.00	7.3	12.7	0.69
	230732	65.0	−13.4	13.4	0.57	1.2	16.1	0.67
	292327	65.6	−3.1	10.0	0.60	11.2	14.0	0.40
	299967	68.3	+8.5	10.0	1.11	4.6	12.3	0.86

^1^ All R^2^ scores significant at 1% level (*p* < 0.05).

## Data Availability

Publicly available datasets were analyzed in this study. These data, all accessed on 16 April 2024, can be found here: PurpleAir (https://www.purpleair.com); OpenAQ (https://openaq.org); AirQino (https://www.airqino.it/en); ARPA Piedmont (https://aria.ambiente.piemonte.it/qualita-aria/dati); ARPA Lombardy (https://www.arpalombardia.it/temi-ambientali/aria/form-richiesta-dati-stazioni-fisse); ARPA Friuli Venezia Giulia (https://www.arpa.fvg.it/temi/temi/aria/sezioni-principali/download-indicatori-e-dati-aria/indicatori-giornalieri-qualita-aria); ARPA Emilia-Romagna (https://sdati-test.datamb.it/arex). Data from ARPA Veneto (https://www.arpa.veneto.it/dati-ambientali/dati-storici/aria/qualita-dellaria-storico-dati-validati) are available on request.

## Supplementary Materials

The following supporting information can be downloaded at: https://www.mdpi.com/article/10.3390/s24123946/s1, Figure S1: Sketch of the AirQino LC air quality monitoring unit; Table S1: Characteristics and pairing of ARPA reference stations used for validation of LC stations in measuring PM_2.5_ daily concentrations; Table S2: Characteristics and pairing of ARPA reference stations used for validation of LC stations in measuring PM_10_ daily concentrations; Table S3: Performance metrics and target values recommended by US EPA for PM_2.5_ air sensors used for non-regulatory supplemental and informational monitoring applications in ambient, outdoor and fixed-site environments: “base” testing [39]; Table S4: Statistics of PM_2.5_/PM_10_ daily concentration ratio measured by LC stations and corresponding paired ARPA reference stations (15 October 2022–15 April 2023); Table S5: Statistical scores of LC stations compared to ARPA reference stations in measuring the PM_2.5_/PM_10_ daily concentration ratio (15 October 2022–15 April 2023).
