# Supplementary material for: Performance Assessment of Two Low-Cost PM2.5 and PM10 Monitoring Networks in the Padana Plain (Italy)
Source: Sensors (Basel). 2024 Jun 18;24(12):3946. doi: 10.3390/s24123946 (PMC11207606; doi:10.3390/s24123946)
Supplement: Supplementary file 1 [file sensors-24-03946-s001.zip › sensors-3029294-supplementary.pdf]

Supplementary material to:

Performance assessment of two low-cost $PM_{2.5}$ and PM_{10} monitoring networks in the Padana Plain (Italy)

Giovanni Gualtieri, Lorenzo Brilli, Federico Carotenuto, Alice Cavaliere, Tommaso Giordano, Simone Putzolu, Carolina Vagnoli, Alessandro Zaldei, and Beniamino Gioli

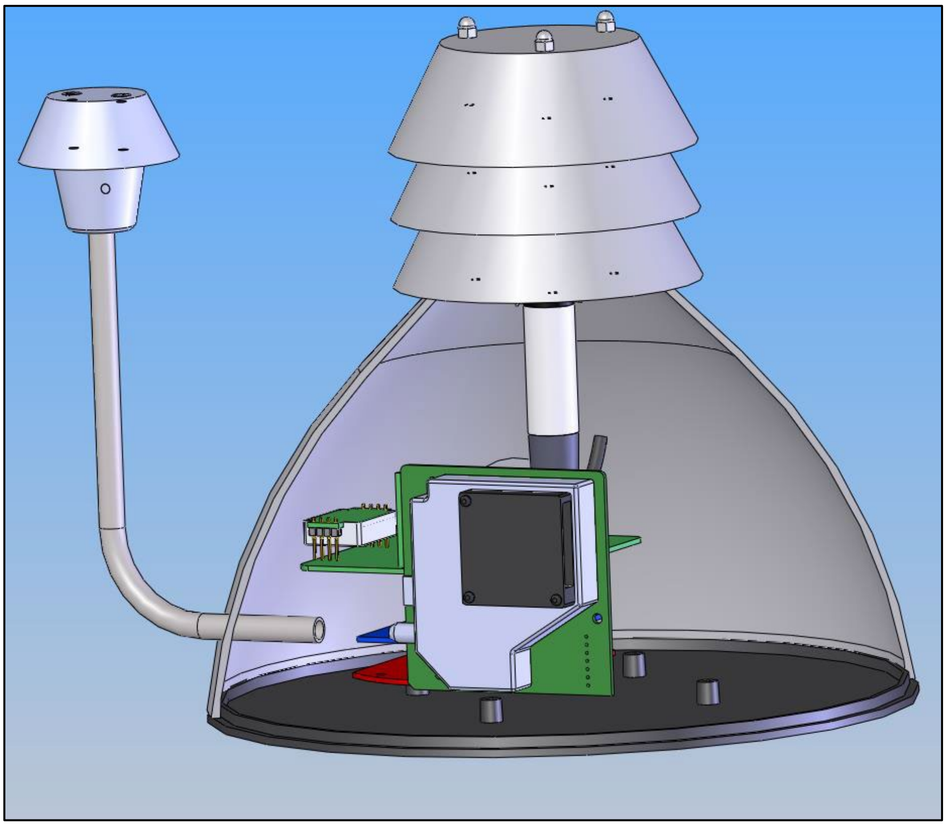

Figure S1. Sketch of the AirQino LC air quality monitoring unit.

Table S1. Characteristics and pairing of ARPA reference stations used for validation of LC stations in measuring $PM_{2.5}$ daily concentrations[1].

ARPA reference stations								LC stations		Distance (km)	Height diff. (m)
European code	Name	Municipality	Region	Type[2]	Latitude (deg N)	Longitude (deg E)	Elevation (m a.s.l.)	ID	Type[2]		
IT0892A	Giardini Margherita	Bologna	Emilia-Romagna	UB	44.4827	11.3541	73	34	RB	8.530	35
IT2243A	Giarol Grande	Verona	Veneto	UB	45.4330	11.0301	47	42	SB	7.580	28
IT2209A	Parco Bertozzi	Faenza	Emilia-Romagna	UB	44.2846	11.8727	38	96	RB	13.500	19
IT1743A	Machiavelli	Monza	Lombardy	UB	45.5811	9.2744	161	132	SB	10.660	3
IT0480A	Sesto San Giovanni	Sesto San Giovanni	Lombardy	UT	45.5348	9.2361	140	135	RT	7.020	19
IT2243A	Giarol Grande	Verona	Veneto	UB	45.4330	11.0301	47	158	RT	8.220	5
IT2168A	Lingotto	Torino	Piedmont	UB	45.0249	7.6491	241	171	UB	1.730	5
IT2120A	Aldo Mei	Beinasco	Piedmont	SB	45.0303	7.5955	263	172	RB	7.470	19
IT2201A	Pordenone Centro 1	Pordenone	Friuli Venezia Giulia	UT	45.9626	12.6559	29	184	SB	2.980	6
IT1737A	Villaggio Sereno	Brescia	Lombardy	UB	45.5153	10.2194	124	64303	UB	1.340	7
IT1975A	Parco Montecucco	Piacenza	Emilia-Romagna	UB	45.0383	9.6692	63	65684	SB	5.460	23
IT1247A	Sturzo	Biella	Piedmont	UB	45.5586	8.0564	402	66626	RB	7.040	19
IT1771A	Parco Ferrari	Modena	Emilia-Romagna	UB	44.6517	10.9264	34	71131	UT	1.890	2
IT1453A	Mandria	Padova	Veneto	UB	45.3719	11.8419	11	229086	UB	5.590	1
IT0912A	Folperti	Pavia	Lombardy	UB	45.1939	9.1647	80	230713	UT	1.920	15
IT0912A	Folperti	Pavia	Lombardy	UB	45.1939	9.1647	80	230729	RB	2.580	7
IT0912A	Folperti	Pavia	Lombardy	UB	45.1939	9.1647	80	230732	RB	1.440	1
IT2168A	Lingotto	Torino	Piedmont	UB	45.0249	7.6491	241	292327	SB	9.820	214
IT1453A	Mandria	Padova	Veneto	UB	45.3719	11.8419	11	299967	SB	8.840	4

[1] Geographic location of ARPA air quality stations is presented in Figure 2.
[2] Station type: UB Urban background; RB, Rural background; SB, Suburban background; UT, Urban traffic; RT, Rural traffic.

Table S2. Characteristics and pairing of ARPA reference stations used for validation of LC stations in measuring PM_{10} daily concentrations[1].

ARPA reference stations								LC stations		Distance (km)	Height diff. (m)
European code	Name	Municipality	Region	Type[2]	Latitude (deg N)	Longitude (deg E)	Elevation (m a.s.l.)	ID	Type[2]		
IT2075A	Chiarini	Bologna	Emilia-Romagna	SB	44.4991	11.2851	45	34	RB	3.940	7
IT2243A	Giarol Grande	Verona	Veneto	UB	45.4330	11.0301	47	42	SB	7.580	28
IT2209A	Parco Bertozzi	Faenza	Emilia-Romagna	UB	44.2846	11.8727	38	96	RB	13.500	19
IT2098A	Monza Parco	Monza	Lombardy	SB	45.6022	9.2761	181	132	SB	11.610	23
IT0477A	Marche	Milano	Lombardy	UT	45.4956	9.1914	126	135	RT	7.540	33
IT1336A	Milano	Verona	Veneto	UT	45.4444	10.9630	62	158	RT	13.610	20
IT2168A	Lingotto	Torino	Piedmont	UB	45.0249	7.6491	241	171	UB	1.730	5
IT2120A	Aldo Mei	Beinasco	Piedmont	SB	45.0303	7.5955	263	172	RB	7.470	19
IT1737A	Villaggio Sereno	Brescia	Lombardy	UB	45.5153	10.2194	124	64303	UB	1.340	7
IT1975A	Parco Montecucco	Piacenza	Emilia-Romagna	UB	45.0383	9.6692	63	65684	SB	5.460	23
IT1246A	Pace	Cossato	Piedmont	UB	45.5689	8.1875	268	66626	RB	4.740	115
IT0721A	Giardini	Modena	Emilia-Romagna	UT	44.6328	10.9036	40	71131	UT	1.380	4
IT1934A	Beccaria	Venezia	Veneto	UB	45.4756	12.2208	2	73781	SB	13.650	12
IT2079A	Valletta	Vigevano	Lombardy	UB	45.3044	8.8458	109	218818	SB	2.340	3
IT1453A	Mandria	Padova	Veneto	UB	45.3719	11.8419	11	229086	UB	5.590	1
IT1104A	Minerva	Pavia	Lombardy	UT	45.1856	9.1469	70	230713	UT	0.330	5
IT0912A	Folperti	Pavia	Lombardy	UB	45.1939	9.1647	80	230729	RB	2.580	7
IT0912A	Folperti	Pavia	Lombardy	UB	45.1939	9.1647	80	230732	RB	1.440	1
IT2168A	Lingotto	Torino	Piedmont	UB	45.0249	7.6491	241	292327	SB	9.820	214
IT1453A	Mandria	Padova	Veneto	UB	45.3719	11.8419	11	299967	SB	8.840	4

[1] Geographic location of LC and ARPA air quality stations is presented in Figure 2.
[2] Station type: UB Urban background; RB, Rural background; SB, Suburban background; UT, Urban traffic; RT, Rural traffic.

Table S3. Performance metrics and target values recommended by US EPA for $PM_{2.5}$ air sensors used for non-regulatory supplemental and informational monitoring applications in ambient, outdoor, fixed site environments: "base" testing [1] [39].

Performance		Target value
**Attribute**	**Metric**	
Precision	Standard deviation	≤ 5 μg/m^3
Bias	Slope	1.0 ± 0.35
	Intercept (b)	-5 ≤ b ≤ 5 μg/m^3
Linearity	Coefficient of determination (R^2)	R^2 ≥ 0.70
Error	Root mean square error (RMSE)	RMSE ≤ 7 μg/m^3

[1] The "base" testing involves field evaluations using 24-hour averaged data performed against a co-located regulatory-grade monitor for at least 30 days of deployment.

Table S4. Statistics of $PM_{2.5}/PM_{10}$ daily concentration ratio measured by LC stations and corresponding paired ARPA reference stations (15/10/2022–15/04/2023).

LC stations						ARPA stations	
Network	ID	Type [1]	Valid data (%) [2]	$PM_{2.5}$ concentrations ($\mu g/m^3$)		$PM_{2.5}$ concentrations ($\mu g/m^3$)	
				Mean	St.dev.	Mean	St.dev.
AirQino	34	RB	90.2	0.84	0.14	0.62	0.19
	42	SB	71.0	0.62	0.12	0.57	0.24
	96	RB	59.6	0.53	0.14	0.59	0.15
	132	SB	80.3	0.58	0.08	0.92	0.58
	135	RT	90.2	0.63	0.10	0.56	0.13
	158	RT	74.3	0.47	0.10	0.58	0.32
	171	UB	85.3	0.54	0.08	0.71	0.11
	172	RB	88.5	0.46	0.06	0.72	0.12
	184	SB	44.3	0.35	0.11	0.67	0.21
	Overall [3]			0.57	0.06	0.67	0.13
PurpleAir	64303	UB	64.5	0.87	0.04	0.72	0.14
	65684	SB	66.7	0.82	0.05	0.76	0.12
	66626	RB	64.5	0.88	0.05	0.54	0.12
	71131	UT	68.3	0.84	0.05	0.58	0.13
	229086	UB	65.6	0.83	0.05	0.80	0.17
	230713	UT	54.6	0.83	0.04	0.73	0.20
	230729	RB	61.2	0.85	0.05	0.75	0.11
	230732	RB	58.5	0.87	0.04	0.75	0.11
	292327	SB	63.9	0.86	0.05	0.73	0.11
	299967	SB	63.9	0.85	0.04	0.80	0.17
	Overall [3]			0.85	0.04	0.72	0.09

[1] Station type: UB Urban background; RB, Rural background; SB, Suburban background; UT, Urban traffic; RT, Rural traffic.

[2] For each LC station and pollutant, valid data refer to the sample of concurrently available LC and ARPA observations for both $PM_{2.5}$ and PM_{10} concentrations.

[3] Overall values by monitoring network of mean concentrations averaged across the full period are based on day-by-day values averaged across all LC or ARPA stations.

Table S5. Statistical scores of LC stations compared to ARPA reference stations in measuring the $PM_{2.5}/PM_{10}$ daily concentration ratio (15/10/2022–15/04/2023).[1]

Network	ID	Valid data (%)	MB	MAE	Slope	Intercept	RMSE	R^2
AirQino	34	90.2	+0.22	0.25	-0.02	+0.85	0.32	0.00
	42	71.0	+0.05	0.17	+0.23	+0.49	0.22	0.20**
	96	59.6	-0.07	0.15	+0.30	+0.35	0.18	0.11**
	132	80.3	-0.35	0.37	+0.04	+0.54	0.66	0.07**
	135	90.2	+0.06	0.10	+0.41	+0.40	0.13	0.28**
	158	74.3	-0.12	0.25	+0.06	+0.43	0.34	0.03*
	171	85.3	-0.17	0.19	-0.16	+0.65	0.23	0.05**
	172	88.5	-0.26	0.27	+0.09	+0.39	0.29	0.03*
	184	44.3	-0.32	0.32	-0.04	+0.38	0.40	0.01
PurpleAir	64303	64.5	+0.15	0.17	-0.13	+0.96	0.22	0.17**
	65684	66.7	+0.06	0.12	-0.07	+0.88	0.15	0.03*
	66626	64.5	+0.34	0.34	-0.15	+0.96	0.37	0.15**
	71131	68.3	+0.26	0.26	-0.07	+0.88	0.30	0.03*
	229086	65.6	+0.03	0.13	+0.00	+0.84	0.18	0.00
	230713	54.6	+0.10	0.16	+0.04	+0.80	0.22	0.04*
	230729	61.2	+0.10	0.13	-0.18	+0.99	0.17	0.13**
	230732	58.5	+0.12	0.14	-0.13	+0.97	0.18	0.12**
	292327	63.9	+0.13	0.14	-0.18	+0.99	0.19	0.14**
	299967	63.9	+0.05	0.12	+0.02	+0.84	0.18	0.00

[1] Significance level for R^2: ** 1% level; * 5% level; ' ' not significant.
